# Discovery and genetic analysis of novel coronaviruses in least horseshoe bats in southwestern China

**DOI:** 10.1038/emi.2016.140

**Published:** 2017-03-29

**Authors:** Lihua Wang, Shihong Fu, Yuxi Cao, Hailin Zhang, Yun Feng, Weihong Yang, Kai Nie, Xuejun Ma, Guodong Liang

**Affiliations:** 1 State Key Laboratory of Infectious Disease Prevention and Control, National Institute for Viral Disease Control and Prevention, Chinese Center for Disease Control and Prevention, Beijing 102206, People's Republic of China; 2 Collaborative Innovation Center for Diagnosis and Treatment of Infectious Diseases, Hangzhou 310003, People's Republic of China; 3 Yunnan Institute of Endemic Diseases Control and Prevention, Yunnan Provincial Key Laboratory for Zoonosis Control and Prevention of Dali, Dali 671000, Yunnan, People's Republic of China

**Keywords:** bat coronavirus, least horseshoe bat, recombination, SARS-CoVs

## Abstract

To investigate bat coronaviruses (CoVs), we collected 132 rectal swabs and urine samples from five bat species in three countries in southwestern China. Seven CoVs belonging to distinct groups of severe acute respiratory syndrome (SARS)-like CoVs and α-CoVs were detected in samples from least horseshoe bats. Samples from other bat species were negative for these viruses, indicating that the least horseshoe bat represents one of the natural reservoirs and mixers for strains of CoVs and has a pivotal role in the evolution and dissemination of these viruses. The genetic and evolutionary characteristics of these strains were described. Whole-genome sequencing of a new isolate (F46) from a rectal swab from a least horseshoe bat showed that it contained 29 699 nucleotides, excluding the poly (A) tail, with 13 open reading frames (ORFs). Phylogenetic and recombination analyses of F46 provided evidence of natural recombination between bat SARS-like CoVs (Rs3367 and LYRa11) or SARS-CoV (BJ01), suggesting that F46 could be a new recombinant virus from SARS-like CoVs or SARS-CoVs.

## INTRODUCTION

The outbreak of severe acute respiratory syndrome (SARS) that emerged in China in 2002–2003 is one of the most significant public health events leading to a global epidemic in the twenty-first century.^1^ SARS caused more than 8000 human cases with 774 deaths.^1, 2, 3^ SARS coronavirus (SARS-CoV) is the etiologic agent of SARS.^4, 5, 6^ Since then, several outbreaks, including avian influenza, Ebola and Middle East respiratory syndrome, have emerged from animal populations causing considerable disease and mortality.^7^ Investigations regarding the direct progenitor and natural reservoirs of SARS-CoVs have demonstrated that bats are the natural reservoir for several viruses that are closely related genetically to SARS-CoV, known as bat SARS-like CoVs.^8, 9^ Bat SARS-like CoVs are thought to be the precursors of SARS-CoV; however, their genomes are not identical to those of SARS-CoV.^10, 11, 12, 13, 14, 15, 16, 17, 18^ The main differences between bat SARS-like CoVs and SARS-CoVs are found in the third non-structural protein (nsp3), spike protein (S), open reading frame (ORF)-3 and ORF-8 regions, which exhibit the lowest nucleotide (nt) and amino-acid (aa) identities among different parts of the genome.^8, 10, 19^ Particularly in the receptor-binding domain (RBD) of S protein, which is responsible for human infection via binding to human angiotensin-converting enzyme 2 (ACE2), bat SARS-like CoVs share only low sequence identity and contain a 19-aa deletion compared with SARS-CoVs.^19, 20, 21^ These differences indicate that the known bat SARS-like CoVs are not the immediate progenitors of SARS-CoVs.^21, 22^

Recently, two bat SARS-like CoVs (Rs3367 and LYRa11) from Chinese horseshoe bats (family: *Rhinolophidae*) were shown to be more closely related to SARS-CoV than any previously identified bat SARS-like CoV. Moreover, these CoVs contain RBDs similar to that in the SARS-CoV S protein for binding to ACE2, suggesting that they can cause direct human infection without an intermediate host.^23, 24^ However, it remains unknown whether bat SARS-like CoVs that are even closer to SARS-CoV exist. Identification of the immediate progenitors of SARS-CoVs and their bat hosts will not only shed light on the animal source of SARS-CoVs but might also have implications in the formulation of strategies to prevent the re-emergence of SARS-like CoVs in human.

Here, we report the genetic and evolutionary characteristics of CoVs identified from least horseshoe bats collected in Yunnan Province, China. In addition, the whole-genome sequence of one isolate was obtained. Genetic, antigenic and recombination analyses of the new isolates were performed.

## MATERIALS AND METHODS

### Ethics statement

Bats were captured, sampled and released according to the guidelines of the Regulations for the Administration of Laboratory Animals (Decree NO 2 of the State Science and Technology Commission of the People's Republic of China, 1988). All experimental protocols were approved by the Ethics Committee of the Institute for Viral Disease Control and Prevention, Chinese Center for Disease Control and Prevention.

### Sample collection

Bats were live-captured with nets in 2012 in three counties/prefectures of Yunnan Province, China. The species were identified and kept in different cages. Clean plastic sheets were placed under bat cages overnight, and urine and fecal samples were collected the following morning (~0730–0800 hours). After sample collection, bats were released at their capture sites. The samples were transported under chilled conditions and stored at −80 °C until being processed.

### Cell culture and virus isolation

Baby hamster kidney (BHK-21, ATCC CCL-10, Manassas, VA, USA) and Tb1Lu (ATCC, CCL88) cell lines were grown in Dulbecco's Modified Eagle Medium containing a high concentration of glucose (Invitrogen, Breda, the Netherlands) and supplemented with penicillin, streptomycin and 10% fetal bovine serum (Invitrogen) at 37 °C in the presence of 5% CO_2_. Urine and fecal samples were thawed at 4 °C and centrifuged at 16 000*g* for 5 min to pellet the debris. The supernatant was filtered through a 0.45-μm filter (Millipore, Darmstadt, Germany) to remove bacterium-sized particles and then diluted 1:10 in cell culture medium. Two 200-μL aliquots of diluted supernatant were added to BHK-21 or Tb1Lu monolayer cells in 24-well plates. After rocking for 2 h at 37 °C, 1 mL of fresh cell culture medium was added, and cells were incubated for seven days at 37 °C. The flasks were observed daily for toxicity, contamination and viral cytopathic effect.

### Reverse transcription-PCR analysis and whole-genome sequencing of F46

Viral RNA was extracted from 140 μL of supernatant from urine and fecal samples using a QIAamp Viral RNA Mini Kit (Qiagen, Germantown, MD, USA) according to the manufacturer's instructions. cDNA was produced using a Ready-To-Go Kit (GE Healthcare, Pittsburg, PA, USA) using random hexanucleotide primers. One-step RT-PCR (reverse transcription-PCR; Invitrogen) was used to detect coronavirus sequences as described previously.^25^ PCR products were gel-purified and cloned into the pGEM-T Easy Vector (Promega, Madison, WI, USA). At least four independent clones were sequenced to obtain a consensus sequence for each of the amplified regions.

Whole-genome sequencing was performed with a next-generation sequencer. Briefly, a Qiagen RNeasy Plus Universal Kit was used to extract the RNA of the F46 isolate from the cell supernatant after one passage in Tb1Lu cells. Genomic DNA was eliminated following the manufacturer's instructions. Reverse transcription, cDNA synthesis and amplification of the cDNA library were conducted using the Nugen RNA-Seq Kit. The Ion OneTouch 2 System was used for template preparation and enrichment. Sequencing was performed using the Ion 318 Chip v2 on the Ion Torrent PGM using barcoded samples. The obtained contigs were subjected to BLAST analysis and assembled using the CLC genomics workbench v. 3.6.5 software (Redwood City, CA, USA). The 5′-rapid amplification of cDNA ends (5′-RACE) and 3′-RACE systems (v. 2.0, Invitrogen) were used to amplify the 5′- and 3′-untranslated regions (UTRs), respectively. To validate the viral genome, we designed primer pairs that generated overlapping amplicons for the whole genome of F46 (primer sequences are available upon request). In addition, the 5' and 3' ends of F46 were confirmed by 5'- and 3'-RACE, respectively.

### Phylogenetic analysis of amplicons

The 405-bp amplicons were aligned with their closest phylogenetic neighbors in the GenBank using ClustalW v.2.0 software (http://www.clustal.org/clustal2/). Representatives of various species in the genera *Alphacoronavirus*, *Betacoronavirus* and *Gammacoronavirus* were included in the alignment. Phylogenetic and molecular evolutionary analyses were performed by the maximum likelihood method using the MEGA v.6 software with the neighbor-joining algorithm and a bootstrap value of 1000.

### Recombination analysis

To detect possible recombination between SARS-CoVs and SARS-like CoVs, the full-length genomic sequence of F46 was aligned with available genome sequences of human/civet (Tor2, AY274119; BJ01, AY278488; SZ3, AY304486; GD01, AY278489; and SZ16, AY304488) and bat SARS-like CoVs (Rp3, DQ071615; Rf1, DQ412042; Rs672, FJ588686; Rm1, DQ412043; Rs3367, KC881006; Cp-Yunnan2011, JX993988; HKU3-1, GQ153542; and LYRa11, KF569997) using ClustalW v.2.0. The aligned sequences were initially scanned for recombination events using the Recombination Detection Program (RDP; version 4) with MaxChi and Chimera methods using 0.6 and 0.05 fractions of variable sites per window, respectively.^25, 26^ The potential recombination events suggested by RDP were investigated further by similarity plot and bootscan analyses using the SimPlot v.3.5.1 software.^26, 27, 28^ Maximum likelihood trees of genomic regions generated by breakpoints were constructed to investigate the phylogenetic origin of parental regions.

### Nt sequence accession numbers

The full genome of F46 and amplicon sequences generated in this study were deposited in GenBank under accession numbers KU973686 to KU973692. The accession numbers of other sequences from GenBank used in this study are indicated in the tables and figure legends.

## RESULTS

### RT-PCR identification of bat alphacoronaviruses and betacoronaviruses

We collected ten *Rhinolophus luctus,* ten *R. affinis* and ten *R. pusillus* at Tengchong; ten *R*. *affinis*, ten *R*. *pusillus* and ten *Myotis daubentonii* at Mangshi; and six fruit bats at Wanding. A total of 66 urine and fecal specimens from the bats, representing various local bat species (Table 1), were collected using plastic sheeting laid in the bat cages in the morning.

**Figure 1:**
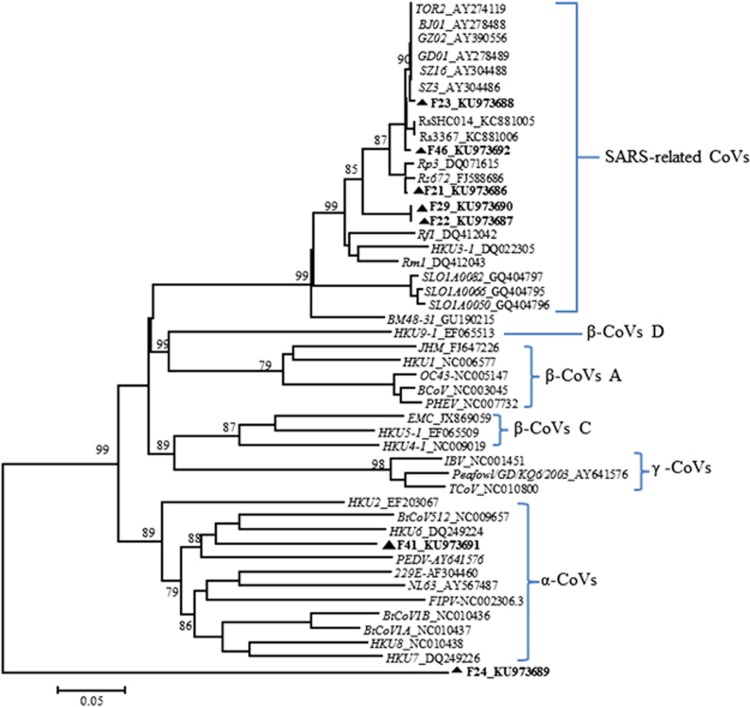
Phylogenetic tree of sequences included in this study and representative CoVs based on partial RdRP gene sequence (405 bp). Analyses were conducted using MEGA v.6 with partial RdRP sequences (405 nt) of each isolate taken from GenBank (corresponding to 15 234–15 638 nt of the genome of bat strain Rp3/2004 (DQ071615) using the neighbor-joining algorithm and bootstrap test for inference of phylogeny. Bootstrap values were obtained from 1000 data sets, and only nodes with bootstrap values greater than 70% are presented. Three established CoV genera, *Alphacoronavirus*, *Betacoronavirus*, and *Gammacoronavirus* are marked as α, β and γ, respectively. Four CoV groups in the genus *Betacoronavirus* are indicated as A, B, C and D. Known human CoVs are in *italics*. Sequences reported in this study are labeled with triangles and highlighted in bold. Coronavirus, CoV; nucleotide, nt.

Seven fecal specimens collected from *R*. *pusillus* at Tengchong and Mangshi were identified with positive bands using nested RT-PCR and were sequenced. The 405-nt partial RNA-dependent RdRP gene sequences (corresponding to nt 15 234–15 638 of the genome of bat strain Rp3/2004, DQ071615) were aligned with those derived from representatives of the three established CoV genera *Alphacoronavirus*, *Betacoronavirus* and *Gammacoronavirus*. Phylogenetic analysis revealed that seven sequences derived from least horseshoe bats were distributed in different clades of the phylogenetic tree. F23 is grouped in one clade of SARS-CoVs and separated from other SARS-like CoVs (SL-CoVs). F46 is grouped in one clade with strain Rs3367 and RsSHC014, which were more closely related to SARS-CoVs than to other SL-CoVs. F21, F22 and F29 are clustered with other SL-CoVs. F41 was grouped with the alphacoronaviruses (α-CoVs). F24 was located in an independent branch. This finding demonstrates great phylogenetic diversity, and the viruses were distributed in relatively separated branches (Figure 1).

### Virus isolation and sequencing of the complete genome

RT-PCR-positive virus isolated from fecal samples was used to inoculate BHK-21 and Tb1Lu cell lines. No obvious viral cytopathic effect was observed after four continuous passages.

Identification of virus isolates was accomplished by RNA extraction and RT-PCR amplification after three freeze–thaw cycles in one to four generations of inoculated BHK-21 and Tb1Lu cells. PCR amplification of sample F46 was positive after the first generation of virus propagation in both cell types but negative in the second through fourth generations after inoculation, indicating that F46 did not propagate well in generations 2–4 of successive cell passages. PCR amplification of other samples in the first through fourth generations of inoculation showed no positive results, suggesting that isolation of potential viruses in these samples failed at the inoculation step in these two cell lines. PCR sequence analysis of the first generation revealed identical sequences with that of the original F46 strain.

F46 RNA was extracted from the cell supernatant after one passage in Tb1Lu cells. Whole-genome sequencing was performed using the Ion Torrent (PGM) Next Generation Sequencer (Life Technologies, South San Francisco, CA, USA), and 16-fold coverage was generated according to the protocol recommended by the manufacturer. A total of 754 Mb of data with 397 895 reads (168–249 bp) was obtained. The CLC genomics workbench was used for sequencing data analysis. Assembly resulted in 450 contigs with lengths from 192 to 3500 bp. Following BLAST and mapping analyses, we obtained 145 viral reads (200–3217 bp) related to CoVs. *De novo* assembly of these sequence reads generated the complete sequences of F46, and no regions had notably lower coverage.

The full length of the F46 genome is 29 699 nt (excluding the poly (A) tail) with a GC content of 41.2%. Its genome organization and coding potential appear similar to SARS-CoV SZ3 with the exception of ORF 4, which is absent from F46 but present in SZ3 (Table 2). The full genome of F46 shared 91.7% to 91.8% nt identity with those of SARS-CoVs. F46 ORFs were compared with human SARS-CoV (Tor2), Civet SARS-CoV (SZ3) and five bat SARS-like CoVs (LYRa11, Rs3367, Rf1, Cp and Rp3; Table 2), and the results indicate that F46 is closely related to SARS-CoVs.

**Figure 2:**
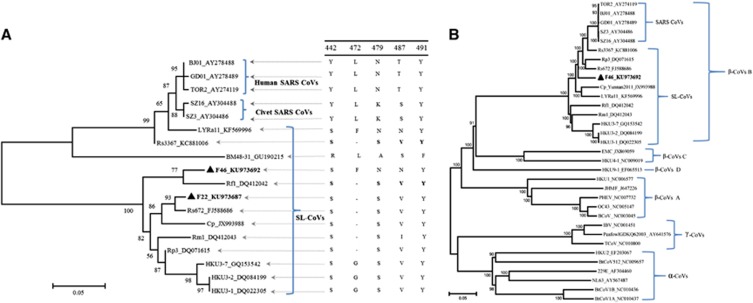
Phylogenetic tree based on the whole-genome sequences and RBD region of F46 and representative coronaviruses. (**A**) Phylogenetic tree based on SARS-CoV S protein amino-acid residues 310–520. Analysis was conducted using MEGA v.6. with a Poisson model. Bootstrap values are determined by 1000 replicates. The key amino-acid residues involved in the interaction with the human ACE2 molecule of identified strains are indicated on the right of the tree. (**B**) Phylogenetic tree based on the whole genome. Analyses were conducted using MEGA v.6 with the neighbor-joining algorithm and bootstrap test for inference of phylogeny. Bootstrap values were obtained from 1000 data sets, and only nodes with bootstrap values greater than 70% are presented. Three established CoV genera *Alphacoronavirus*, *Betacoronavirus* and *Gammacoronavirus* are marked as α, β, and γ, respectively. Four CoV groups in the genus *Betacoronavirus* are indicated as A, B, C and D. Angiotensin-converting enzyme 2, ACE2; coronavirus, CoV; receptor-binding domain, RBD; severe acute respiratory syndrome, SARS.

Phylogenetic analysis based on the whole genome of F46 and representatives of three established coronavirus genera (*Alphacoronavirus*, *Betacoronavirus* and *Gammacoronavirus*), including the human/civet and bat SARS-like CoVs, demonstrated that F46 clusters with bat SARS-like CoVs (Figure 2B). The location of F46 in the phylogenetic tree is between LYRa11 and Rs3367, but they are distributed in relatively separate clades. F46 likely represents an intermediate between LYRa11 and Rs3367 in the evolutionary pathway. Further verification of this finding is presented below in the recombination analysis.

### Genetic and antigenic characterization of the spike protein of F46

**Figure 3:**
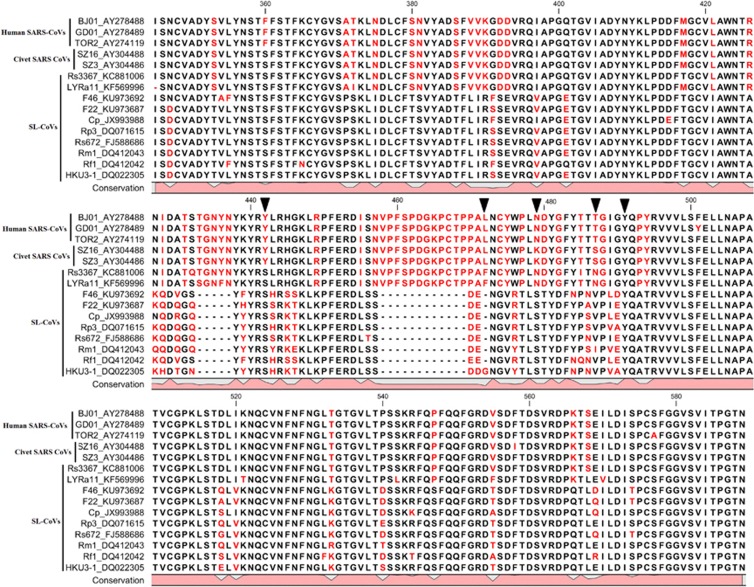
Sequence alignment of CoV S protein RBD. The RBD of S protein from F46, Human/Civet CoVs and other closely related bat SARS-like CoVs were aligned using ClustalW 2.0. The key amino-acid residues involved in the interaction with human ACE2 are indicated by arrows. Angiotensin-converting enzyme 2, ACE2; coronavirus, CoV; open reading frame, ORF; receptor-binding domain, RBD; severe acute respiratory syndrome, SARS.

The spike (S) proteins of CoVs are responsible for receptor-binding and host species adaptation, and their genes constitute one of the most variable regions within CoV genomes. Similar to counterparts in other SARS-CoVs or SARS-like CoVs, the S gene of F46 exhibited wide variation, sharing 76.3%–76.5% aa identity with SARS-CoVs and 76.5%–83.5% aa identity with bat SARS-like CoVs (Table 2). Spike protein is post-translationally cleaved into S1 and S2 subunits. The S1 domain of SARS-CoV S protein mediates virus binding with ACE2, the functional receptor for SARS-CoV on susceptible cells. The RBD of S1 mediates receptor binding of the virus to host cells and determines the host spectrum.^29, 30^ RBD aa sequence comparisons among F46, human/civet viruses and bat SARS-like CoVs revealed that F46 shares 76.1% to 76.5% identity with human/civet SARS-CoVs and 76.5% to 83.5% identity with bat SARS-like CoVs. The S2 domain of F46 exhibited less diversity between F46 and other bat SARS-like CoVs or SARS-CoVs. S2 domain of F46 shares 94.5% to 95.0% aa identity with human/civet SARS-CoVs and 93.7% to 98.3% aa identity with bat SARS-like CoVs. F46 has three aa deletions: 432–436 aa, 457–460 aa and 464–472 aa compared with human/civet CoVs and bat SARS-like CoVs LYRa11 and Rs3367. Of the five key aa residues (442, 472, 479, 487 and 491) involved in human ACE2 receptor recognition and enhancement of receptor binding, three mutations (Y442S, N479S and T487V) were observed in F46 compared with SARS-CoVs (Figures 2A and 3).^31, 32^

### Recombination analysis

**Figure 4:**
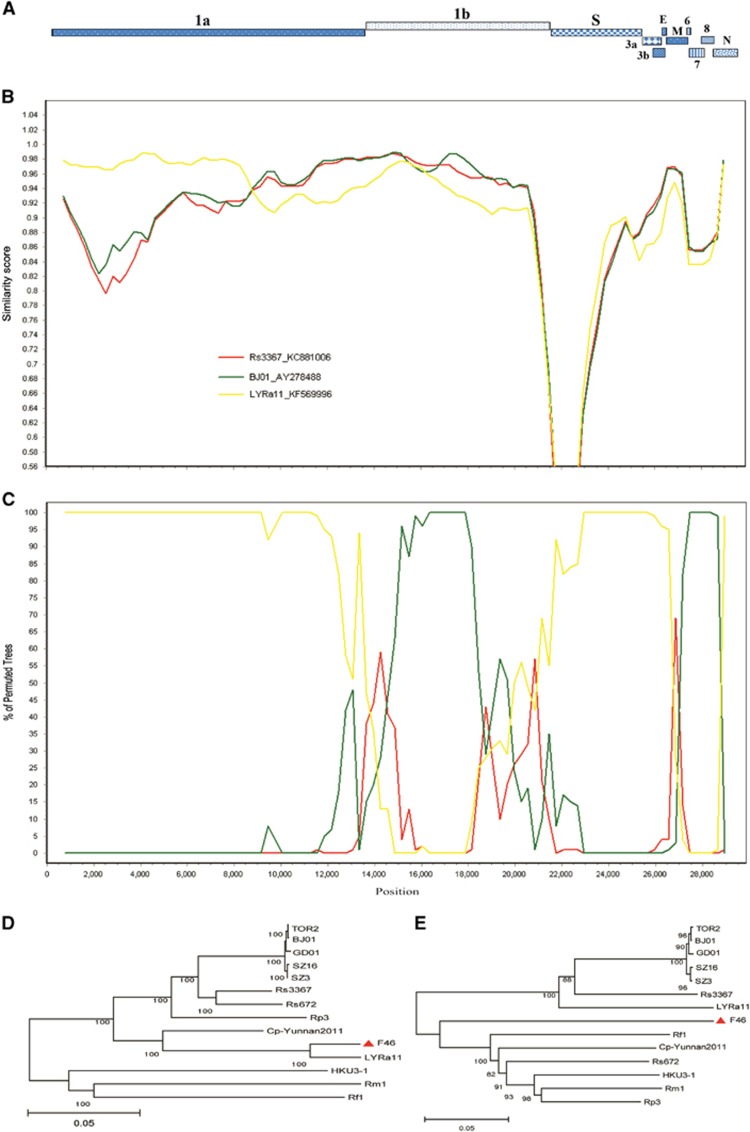
Recombination analysis of F46 with SARS-CoVs and SL-CoVs. (**A**) A gene map of LYRa11 is used to position breakpoints. Four breakpoints were detected in the F46 genome with strong *P*-values (<10^–30^) supported by similarity plot and bootscan analyses (**B, C**). The breakpoints generated recombination of fragment A covering 1–8 and 848 nts (a total of 8 and 848 nt), including the 5′-UTR and partial ORF 1a, and fragment B covering 21 684 to 23 271 nt (a total of 1587 nt), including a partial ORF of the spike gene. Similarity plots (**B**) and bootscan analyses (**C**) were conducted with F46 as the query and bat SARS-like CoVs (Rs3367 and LYRa11) and SARS-CoV BJ01 as potential parental sequences. Phylogenetic trees (**D, E**) were constructed based on the two fragments with a neighbor-joining algorithm and a bootstrap test for inference of phylogeny. Bootstrap values were obtained from 1000 data sets. Coronavirus, CoV; nucleotide, nt; open reading frame, ORF; severe acute respiratory syndrome, SARS; untranslated region, UTR.

CoVs commonly undergo RNA recombination between strains at high frequency. To understand the evolutionary origin and identify the recombination events of F46, we conducted a recombination analysis using RDP and available genome sequences of human/civet and bat SARS-like CoVs. Four breakpoints were detected in the F46 genome, with strong *P*-values (<10^–30^) supported by similarity plot and bootscan analyses (Figures 4B and 4C). The breakpoints generated recombination of fragment A covering 1–8 and 848 nts (a total of 8 and 848 nt), including the 5′-UTR and partial ORF 1a, and fragment B covering 21 684 to 23 271 nt (a total of 1587 nt), including a partial ORF of the spike gene. Both similarity plots and bootscan analyses revealed that F46 was highly phylogenetically related to SL-CoV LYRa11 in the first 9000 nt of the genome. Downstream of the first 9000 nt of the genome, F46 shared similar sequence similarity to SARS-CoV BJ01 and SL-CoV Rs3367. Phylogenetic analysis using the major and minor parental regions showed that F46 is grouped in the SL-CoVs and close to SL-CoV LYRa11(Figures 4D and 4E). These results indicate that F46 could be a new recombinant virus from SARS-like CoVs or SARS-CoVs.

## DISCUSSION

SARS and Middle East respiratory syndrome-CoV are two novel CoVs discovered in recent years that cause considerable disease and mortality.^1, 2, 5, 33, 34^ The origin of the viruses and the extent of their involvement in both human and animal populations remain hot topics.^33, 34, 35, 36, 37^ It is believed that Middle East respiratory syndrome-CoV, like many other CoVs, originated in bats.^33, 34^ This hypothesis proved to be true with SARS based on several studies.^35, 36, 37, 38^ Recent metagenomics studies have identified sequences of closely related SARS-like viruses circulating in Chinese bat populations that may pose a future threat (references). In 2005, two independent studies identified SARS-like CoVs from bats.^20, 33^ Since then, additional SARS-like CoVs in various bat species have been reported.^24, 25^ Among them, horseshoe bats (*Rhinolophus*) are the main reservoir to harbor genetically diverse SARS-like CoVs.^10, 11, 13, 15, 17, 19, 20, 23, 24, 25^ Several of these SARS-like CoVs, such as Rs3367 and LYRa1, appear to be most closely related to SARS-CoVs and the gap-filling viruses between bat SARS-like CoVs and human SARS-CoVs.^23, 24^ In this study, several CoVs belonging to the groups of SARS-like CoVs and α-CoVs were detected in samples from least horseshoe bats (*R*. *pusillus*). Samples from other bat species (*R*. *luctus*, *R*. *affinis*, *M*. *daubentonii* and fruit bat) were negative for these viruses, further confirming that the least horseshoe bat is a prominent natural reservoir or mixer of genetically diverse *Alphacoronavirus* and *Betacoronavirus* species and plays a pivotal role in the evolution and dissemination of these viruses.

Although we failed to isolate infectious virus from the collected samples, we obtained the complete genome sequence of F46 from the Tb1Lu cell line culture after one passage. The S protein RBD of F46 possesses identifying features typical of those belonging to bat SARS-like CoVs, namely three deletions and mutations within the key residues required for binding ACE2 (Figures 2A and 3).^31, 32^ Predicting from these features, F46 should be incapable of infecting humans directly. Phylogenetic analysis demonstrates that F46 is placed in one clade with strain Rs3367 and RsSHC014, which were more closely related to SARS-CoVs than to other SL-CoVs (Figure 1), and particularly between LYRa11 and Rs3367 (Figure 2). F46 likely represents an intermediate between LYRa11 and Rs3367. To understand the evolutionary pathway of F46, we conducted recombination analysis using available genome sequences of human/civet and bat SARS-like CoVs. A total of four breakpoints were detected in the F46 genome with strong *P*-values (<10^–30^). The results showed that F46 is a recombinant from Rs3367, LYRa11 and BJ01 lineages.

In conclusion, horseshoe bats carry genetically diverse SARS-like CoVs. Owing to the high likelihood of recombination among bat CoVs, additional bat SARS-like CoVs are likely to be identified in the future. To better predict and prevent the next emergence of disease caused by CoVs of bat origin, it is necessary to maintain long-term surveillance of bat CoVs.

## Figures and Tables

**Table 1:** Information of collected samples

**Location**	**Species**	**Urine**	**Feces**
Tengchong	*Rhinolophus luctus*	U1–U10	F1–F10
	*Rhinolophus affinis*	U11–U20	F11–F20
	*Rhinolophus pusillus*	U21–U30	F21–F30
Mangshi	*Rhinolophus affinis*	U31–U40	F31–F40
	*Rhinolophus pusillus*	U41–U50	F41–F50
	*Myotis daubentonii*	U51–U60	F51–F60
Wanding	Fruit bats	U61–U66	F61–F66

**Table 2:** Comparison of full-genome lengths, ORF and aa identities of F46 with SARS and bat SARS-like CoVs

**Virus strains**	**F46**	**LYRa11**	**Rs3367**	**Rf1**	**Cp**	**Rp3**	**Tor2**	**SZ3**
**Origin**	***Rhinolophus pusillus***	***Rhinolophus affinis***	***Rhinolophus sinicus***	***Rhinolophus ferrumequinum***	***Chaerephon plicata***	***Rhinolophus pearsoni***	**SARS patient**	**Palm Civet**
Genome (bases)	29 722	29 805	29 792	29 709	29 452	29 736	29 751	29 741
G+C (%)	41.2	40.7	40.8	41.1	40.9	40.9	40.8	40.8
*ORF (length, aa/%aa identity)*								
1a	4382	**4382/98.5**	4382/95.2	4377/93.1	4382/96.7	4380/94.1	4377/95.6	4382/95.7
1b	2628	2628/99.1	**2628/99.3**	2626/98.4	2628/98.7	2626/99.2	**2641/99.3**	**2628/99.3**
S	1236	1259/77.7	1256/76.5	1241/81.0	1241/83.1	**1241/83.5**	1255/76.5	1255/76.3
3a	274	274/82.1	274/87.9	274/89.4	**274/94.1**	274/88.3	274/86.8	274/86.4
3b	NP	NP/NA	114/NA	114/NA	NP/NA	NP/NA	154/NA	154/NA
E	76	**76/100**	**76/100**	76/96.0	**76/100**	**76/100**	**76/100**	**76/100**
M	221	221/96.3	**221/99.5**	221/99.0	221/97.7	221/98.6	221/98.6	221/98.1
6	63	63/92.0	63/93.6	63/93.6	63/95.2	63/92.0	**63/96.8**	**63/96.8**
7a	122	122/92.6	122/95.9	122/95.9	122/95.9	122/95.0	**122/96.7**	**122/96.7**
7b	44	44/88.6	44/97.7	44/95.4	44/88.6	**44/97.7**	44/90.9	44/90.9
8a	122	NP	NP	122/79.2	NP	NP	39/27.0	**122/95.0**
8b	NP	NP/NA	NP/NA	NP/NA	NP/NA	NP/NA	84	NP/NA
N	422	**422/98.8**	422/98.5	421/95.2	422/98.5	421/97.6	422/98.5	422/98.5
9b	98	98/95.9	**98/97.9**	97/83.6	98/94.8	97/87.7	**98/97.9**	**98/97.9**

Abbreviations: amino acid, aa; coronavirus, CoV; not available, NA; not present, NP; open reading frame, ORF; severe acute respiratory syndrome, SARS.

Note: The accession numbers of F46, LyRa11, Rs3367, Rf1, Cp, Rp3, Tor2 and SZ3 are KU973692, KF569996, KC881006, DQ412042, JX993988, DQ071615, AY274119 and AY304486, respectively. The % identity shows aa sequence identity with F46. The highest % identities are highlighted.
